# Real‐Life Cohort of Patients With Resected High‐Risk Melanoma Treated by Adjuvant Anti‐PD1 Therapy

**DOI:** 10.1002/cam4.70432

**Published:** 2025-03-19

**Authors:** Liza Benzoni, Anaïs Eberhardt, Sarah Milley, Safa Idoudi, Camille Trefcon, Nicolas Romain‐Scelle, Luc Thomas, Stéphane Dalle

**Affiliations:** ^1^ Service de Dermatologie, Hospices Civils de Lyon Hôpital Lyon Sud Lyon France; ^2^ Centre Léon Bérard, Cancer Research Center of Lyon, INSERM 1052, CNRS 5286 Université Claude Bernard Lyon 1, Université de Lyon Lyon France; ^3^ Université Claude Bernard Lyon 1 Lyon France; ^4^ Service de Dermatologie, Hôpital Saint Louis Paris France; ^5^ Service de Biostatistique, Hospices Civils de Lyon Hôpital Lyon Sud Pierre‐Bénite France

**Keywords:** adjuvant, anti‐PD1 therapy, high‐risk melanoma

## Abstract

**Background:**

Programmed cell death protein‐1 (PD1) antibodies are currently the standard treatment for resected high‐risk melanoma, yet recurrence rate remains high.

**Objectives:**

This real‐life observational study aimed to describe the outcomes of patients with resected high‐risk melanoma following adjuvant anti‐PD1 immunotherapy and identify factors associated with recurrence risk.

**Materials and Methods:**

A total of 235 patients with resected stage III/IV melanoma treated with adjuvant nivolumab or pembrolizumab were included. Imaging scans and cerebral imaging were performed every 12 weeks to detect recurrences. Adverse events were collected. Univariate and multivariate analyses were performed to identify predictive factors of recurrence. Overall survival (OS) and recurrence‐free survival (RFS) were estimated.

**Results:**

Among the 235 patients, 103 experienced at least one recurrence (43%); first recurrences were predominantly locoregional (47%). The predictive factor for recurrence identified by multivariate analysis was ulceration (RR 2,03, 95% CI [1,20; 2,86]). RFS was estimated at 75% [70–81] at 12 months and at 64% [58–71] at 24 months. RFS at 12 months was significantly lower in patients with ulcerations (RFS at 83%) compared to those without ulceration (RFS at 66%), *p* < 0.01. Overall survival (OS) was estimated at 91% [87%–94%] at 12 months and 84% [79%–89%] at 24 months. The OS after a first recurrence was estimated at 69% [60%–80%] at 12 months and decreased to 43% [32%–57%] at 24 months. After a first locoregional recurrence, surgery with a year of adjuvant immunotherapy (40%) was the favoured therapeutic approach. For distant recurrences, clinical trial enrolment was preferred (21%). Double curative immunotherapy was the preferred strategy for cerebral recurrences (30%).

**Conclusions:**

In this cohort, nearly half of the patients underwent recurrences and RFS at 24 months was 64%. The RFS and OS data were comparable o those reported in the pivotal study Ulceration was the only significant predictive factor for recurrence, associated with decreased RFS at 24 months.

## Graphical abstract

This real‐life study based on 235 patients treated with adjuvant anti‐PD1 therapy provides a comprehensive view of the outcomes and factors associated with recurrence in patients with resected high‐risk stage III/IV melanoma. Ulceration emerged as the only significant predictive factor for recurrence and was associated with a decrease in recurrence‐free survival at 24 months.

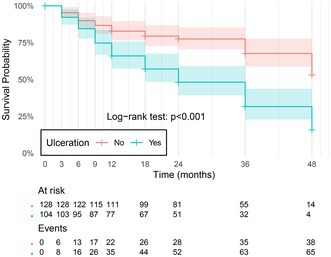

## 1 Introduction

The effectiveness of immunotherapy, using immune checkpoint inhibitors (ICI) such as anti‐PD1 and anti‐CTLA‐4 antibodies, was initially demonstrated in the treatment of metastatic melanoma [1, 2], thereby transforming the management and prognosis of these patients. Subsequently, its application as adjuvant was explored in two randomised trials [3, 4]. In the EORTC 1325/KEYNOTE‐054 study, a 12‐month recurrence‐free survival (RFS) of 75.4% [95% CI: 71.3–78.9] was observed in patients with resected stage III and IV melanoma treated with pembrolizumab, compared to 61.0% [95% CI: 56.5–65.1] in the placebo group. This improvement persisted over time, with a RFS of 63.7% in the pembrolizumab group compared to 44.1% in the placebo group after 3 years of follow‐up [3, 5, 6]. The CheckMate 238 study compared nivolumab to ipilimumab and found a 12‐month RFS of 70.5% [95% CI: 66.1–74.5] in the nivolumab group versus 60.8% [95% CI: 56.0–65.2] in the ipilimumab group, with fewer grade 3 or 4 adverse events in the nivolumab group (14.4% vs. 45.9% in the ipilimumab group) [4, 7, 8].

Consequently, adjuvant immunotherapy became a new standard of care for resected stage III and IV melanoma by significantly improving RFS. However, approximately a third of patients still experience recurrences following adjuvant immunotherapy, and the management of these recurrences is not yet standardised. To date, limited data are available regarding patients receiving adjuvant immunotherapy outside the setting of clinical trials. The characteristics distinguishing recurring patients from non‐recurring ones, as well as their outcomes after recurrence, remain uncertain. The primary objective of this real‐life observational study was to describe the outcomes of patients with resected high‐risk melanoma following adjuvant anti‐PD1 immunotherapy. The secondary objective was to identify factors associated with a risk of recurrence in this population.

## 2 Materials and Methods

We retrospectively and consecutively included all patients who received at least one course of adjuvant anti‐PD1 therapy for resected stage III and IV melanoma in the tertiary care centre dermatology department of the *Hôpital Lyon Sud* (Pierre‐Bénite, France) between the 1st of January 2019 and the 31st of December 2021. The data concerning patient follow‐up were collected until August 31, 2023. All included patients were adults and received their first infusion of pembrolizumab or nivolumab within 12 weeks of the surgical intervention. Patient staging was performed according to the criteria of the 8th edition of the American Joint Committee on Cancer (AJCC).

Comprehensive evaluations, including imaging scans and cerebral imaging (brain CT or MRI in case of suggestive symptoms or confirmed brain lesions), were conducted every 12 weeks to detect recurrences. Simultaneously, clinical assessments were performed every 4 or 6 weeks, depending on the infusion frequency. Locoregional recurrences were defined by the presence of cutaneous, subcutaneous or lymph node metastases between the primary melanoma site and the first lymph node relay. Distant recurrences were characterised by the presence of distant metastases without cerebral involvement. Cerebral recurrences were defined by the presence of cerebral metastases with or without distant metastases. Adverse events were assessed and graded according to the National Cancer Institute Common Terminology Criteria for Adverse Events (NCI CTCAE), version 5.0.

Statistical analyses were performed by independent biostatisticians. The univariate analysis was conducted using the Wilcoxon rank‐sum test for continuous data and both the Fisher's exact test and the Pearson's chi‐squared test (whichever was appropriate) for categorical data. Nine variables were tested for association with the probability of one or more recurrences in 48 months, selected from clinical expertise: age, Breslow thickness, localisation of primary tumour, mutational status, number of initial mitoses, ulceration status, type of initial involvement and history of inflammatory disease. Variables for which the p‐value was under 0.2 in univariate analysis were included in a single multivariate logistic regression, and final relevance was evaluated using a likelihood ratio test. Survival analyses estimating overall survival (OS) and RFS were conducted using the Kaplan–Meier method and the log‐rank test. The significance threshold was set at a *p* < 0.05. The analysis was conducted in R, using the *marginal effects* package for the relative risks' computation [9].

The study was approved by an ethics committee on the 9th of September 2022 (opinion n°22_616). The collection and use of personal data followed the French Reference Methodology MR‐004 of the national data protection agency (*Commission Nationale de l'Informatique et des Libertés*, CNIL).

## 3 Results

### 3.1 Patient Characteristics

A total of 235 patients were included; their mean age was 60 (SD 16) years and 55% were male. The majority of patients presented with cutaneous or acral melanoma (211 patients, 90%), while only 6 (2%) had mucosal melanoma and 18 (8%) had a primary melanoma of unknown location. The mean (SD) Breslow thickness was 4.5 (4.2) mm. Ulceration on the primary melanoma was present in 45% of the cases, absent in 39% and unknown in 16%. Mitotic status was low (1 to 3 mitoses/mm^2^) in 28% of the patients, moderate (4 to 10 mitoses/mm^2^) in 34% and high (more than 10 mitoses/mm^2^) in 15% (Table 1).

**TABLE 1:** Patient and tumour characteristics at study inclusion.

Characteristics	*N* = 235
Sex, *n* (%)	
Male	129 (55)
Female	106 (45)
Age at first infusion, mean (SD)	60 (16)
Treatment administered, *n* (%)	
Nivolumab	154 (66)
Pembrolizumab	81 (34)
Localisation of primary tumour, *n* (%)	
Cutaneous or acral	211 (90)
Mucosal	6 (2)
Unknown	18 (8)
Breslow thickness (mm), mean (SD)	4.5 (4.2)
Unknown	24
Mutational status, *n* (%)	
No mutation	74 (31)
BRAF V600 mutation alone	103 (44)
NRAS mutation alone	52 (22)
BRAF V600 and NRAS mutations	4 (2)
unknown	2 (1)
Mitoses, *n* (%)	
None	16 (7)
[1; 3]	66 (28)
[4; 10]	80 (34)
> 10	36 (15)
Unknown	37 (16)
Ulceration of primary tumour, *n* (%)	
Absent	93 (39)
Present Unknown	106 (45) 36 (15)
Unknown	36 (16)
Inflammatory disease history, *n* (%)	
None	212 (90)
Present	23 (10)
Type of involvement at treatment initiation, *n* (%)	
Clinically occult nodal metastasis	120 (51)
Clinically detected nodal metastasis	75 (32)
In‐transit metastasis	16 (7)
Distant metastases resected	24 (10)
AJCC stage, *n* (%)	
IIIA	28 (12)
IIIB	68 (29)
IIIC	111 (47)
IIID	4 (2)
IV	24 (10)
Onset of vitiligo *n* (%)	
Yes	6 (4.3)
No	229 (96)
Reason for treatment discontinuation, *n* (%)	
Scheduled end of treatment	139 (59)
Recurrence	68 (29)
Death	3 (1)
Toxicity	21 (9)
Patient's decision	5 (2)
Type of recurrence, *n* (%)	
Locoregional	49 (47)
Distant without cerebral involvement	44 (43)
Cerebral with or without distant metastases metamereinvolvement	10 (10)
Time of recurrence, *n* (%)	
During treatment	73 (71)
After end of treatment	30 (29)

The highest proportion of patient (44%) had an isolated BRAF gene mutation, while 22% had an isolated NRAS gene mutation. An absence of BRAF and NRAS mutations was observed in 31% of the patients, and only 2% of the patients had a combination of BRAF and NRAS mutations.

Regarding the initial stage at the time of treatment initiation, 90% of the patients were classified as stage III: 12% were classified as stage IIIA, 29% as stage IIIB, 47% as stage IIIC, and only 2% as stage IIID. Among stage III patients, most had clinically occult nodal metastases (51%), while 32% had clinically detected nodal metastases and 7% had in‐transit metastases. The remaining patients (10%) presented with resected stage IV melanoma.

Overall, 66% of patients were treated with nivolumab while the remaining 34% were treated with pembrolizumab. An underlying inflammatory or autoimmune disease was present in 23 patients at the time of treatment initiation, representing 10% of the population. The median (IQR) follow‐up time was 24 (15–33) months.

### 3.2 Adverse Events

A total of 143 (61%) patients experienced an adverse event during treatment (Table 2). Only 1 patient experienced an immune‐mediated adverse event after the completion of immunotherapy. Among all adverse events, the majority were grade 1 and 2 (42% and 44%, respectively), while 10% of the patients experienced a grade 3 adverse event and 2% experienced a grade 4. Most patients presented with only one adverse event during monitoring (83%) while 16% reported 2 different events. Only one death was reported as treatment‐related and consisted of a severe immune‐mediated diabetes with ketoacidosis in an 86‐year‐old woman. The occurrence of vitiligo was reported in 6 patients.

**TABLE 2:** Description of treatment‐related adverse events.

	*N*
Adverse events, *n* (%)	*N* = 235
None reported	91 (39)
During treatment	143 (61)
After the end of treatment	1 (0.4)
AE grade	*N* = 153
1	65 (42)
2	68 (44)
3	16 (10)
4	3 (2)
5	1 (0.7)
Highest grade of AE presented, *n* (%)	*N* = 130
1	50 (38)
2	61 (47)
3	15 (12)
4	3 (2)
5	1 (0.7)
Number of AE by patient, *n* (%)	*N* = 130
1	108 (83)
2	21 (16)
3	1 (0.7)
Onset of vitiligo, *n* (%)	*N* = 235
Yes	6 (4.3)
No	229 (96)

### 3.3 Recurrences

**FIGURE 1:**
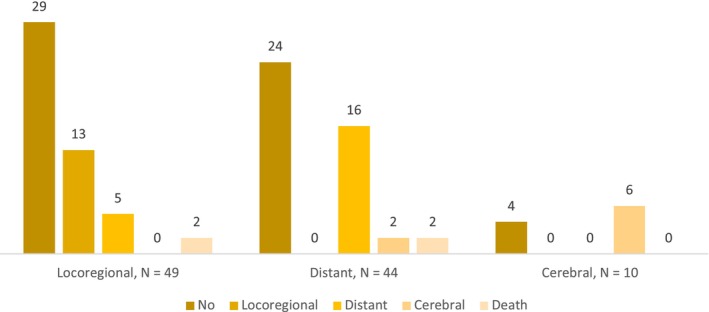
Proportion and localisation of second recurrences according to the site of first recurrence.

Overall, 59% of the patients completed the full year of adjuvant immunotherapy. In case of early discontinuation, the main reason was recurrence (29%). Treatment toxicity (9%) was the second cause of immunotherapy discontinuation and 2% of the patients stopped treatment due to their personal decision. Overall, 43% of the patients experienced a recurrence, which occurred during treatment in 71% of the cases. The most common site of first recurrence was locoregional in 47% of the cases. A distant recurrence without cerebral involvement occurred in 43% of the patients and 10% experienced a cerebral recurrence with or without distant metastases. After an initial locoregional recurrence, 27% of the patients experienced a second locoregional recurrence, 10% had a second distant recurrence, and 2 patients died. In patients for whom the first recurrence was distant without cerebral involvement, 36% had a second distant recurrence, 4.5% experienced a second cerebral recurrence, and 2 patients died. Among the patients with a first cerebral recurrence, 60% had a second cerebral recurrence, while the remaining 4 patients did not experience a second recurrence (Figure 1).

### 3.4 Second‐Line Therapy

In cases where the first recurrence was locoregional, the most frequently adopted approach was surgical management followed by the resumption of adjuvant immunotherapy for a year (40%). For patients with a BRAF mutation, the option of introducing anti‐BRAF anti‐MEK targeted therapy after surgery was considered in 15% of the cases. For 13% of the patients, an extension of simple immunotherapy for curative purposes was chosen. Double immunotherapy was suggested for one specific patient with vulvar melanoma who presented multiple nodules in the vulvar region. Chemotherapy was performed in 11% of the patients and 6% of the patients were included in clinical trials. Overall, 8.5% of the patients opted for alternative treatments, such as topical imiquimod and surgery or neo‐adjuvant immunotherapy followed by subsequent surgery. Only 1 patient refused to undergo systemic treatment and was treated by surgery alone.

In patients who had experienced a first distant recurrence without cerebral involvement, enrolment in a clinical trial was the preferred option (21%). Continuing immunotherapy for curative purposes and chemotherapy were proposed to 16% of the patients in each case. When the distant recurrence involved a single metastasis, the proposed approach was surgical intervention combined with adjuvant immunotherapy (14%).

Among the 10 patients for whom the first recurrence was cerebral, 3 patients were treated with double immunotherapy, and 3 were treated with targeted therapy, including 1 associated with radiotherapy. One patient was treated with radiotherapy combined with immunotherapy and another with chemotherapy. Finally, 1 patient was enrolled in a clinical trial and 1 underwent palliative care only.

### 3.5 Predictive Factors of Recurrence

In univariate analysis, no significant differences were found between patients who presented a recurrence and those who did not in terms of treatment, mutational status, number of initial mitoses, type of initial involvement and history of inflammatory disease. There was a significant difference regarding age, Breslow thickness and the presence of ulceration between patients with and without recurrence (Table 3).

**TABLE 3:** Univariate analysis of factors associated with recurrence‐free survival in patients treated with adjuvant anti‐PD1 therapy.

Variable	*N*	Total population *N* = 235	Patients without progression *N* = 129	Patients with progression* *N* = 106	*p*
Age, median (IQR)	235	63 (23)	60 (23)	68 (21)	0.013^1^
Breslow, median (IQR)	211	3.0 (3.5)	3.0 (2.4)	3.7 (4.9)	0.006^1^
Unknown		24	16	8	
Localisation of primary tumour, *n* (%)	217				0.10^2^
Cutaneous or acral		211 (97)	116 (99)	95 (95)	
Mucosal		6 (2.8)	1 (0.9)	5 (5.0)	
Unknown		18	12	6	
Treatment administered, *n* (%)	235				0.90^3^
Nivolumab		154 (66)	85 (66)	69 (65)	
Pembrolizumab		81 (34)	44 (34)	37 (35)	
Mutational status, *n* (%)	235				0.63^4^
No mutation		74 (31)	42 (33)	32 (30)	
BRAF V 600 mutation alone		103 (44)	59 (46)	44 (42)	
NRAS mutation alone		52 (22)	24 (19)	28 (26)	
BRAF V 600 and NRAS mutations		4 (1.7)	3 (2.3)	1 (0.9)	
Unknown		2 (0.9)	1 (0.8)	1 (0.9)	
Mitoses, *n* (%)	198				0.16^3^
0		16 (8.1)	9 (8.5)	7 (7.6)	
[1; 3]		66 (33)	41 (39)	25 (27)	
[4; 10]		80 (40)	42 (40)	38 (41)	
> 10		36 (18)	14 (13)	22 (24)	
Unknown		37	23	14	
Ulceration of primary tumour, *n* (%)	235				< 0.001^3^
Absent		129 (55)	90 (70)	39 (37)	
Present		106 (45)	39 (30)	67 (63)	
Inflammatory disease history, *n* (%)	235				0.87^3^
0		212 (90)	116 (90)	96 (91)	
1		23 (9.8)	13 (10)	10 (9.4)	
Type of involvement at treatment initiation, *n* (%)	235				0.82^3^
Clinically occult nodal metastasis		120 (51)	65 (50)	55 (52)	
Clinically detected nodal metastasis		75 (32)	40 (31)	35 (33)	
In‐transit metastases		20 (8.5)	11 (8.5)	9 (8.5)	
Distant metastases resected		20 (8.5)	13 (10)	7 (6.6)	

^1^ Wilcoxon rank sum test.

^2^ Fisher's exact test.

^3^ Pearson's Chi‐squared test.

^4^ Fisher's exact test for count data with simulated *p*‐value (based on 1000 replicates).

* The patients with progression considered were those who presented at least one recurrence (*n* = 103) as well as those who died before a first recurrence (*n* = 3).

In multivariate analysis, the risk of recurrence was multiplied by 2.03 (95% CI: [1.20; 2.86]) in patients with ulcerated melanomas. No other candidate predictors were found to be significantly associated with the risk of recurrence (Table 4).

**TABLE 4:** Multivariate analysis with *p*‐value associated with log‐likelihood test for each variable.

Variable	Contrast	Risk ratio	95% CI	*p* (ANOVA)
Age	+1 year	1.00	[0.99; 1.01]	0.5
Breslow thickness	+1 mm	1.03	[0.98; 1.07]	0.2
Number of mitosis [1; 3]	[1; 3] versus 0	0.77	[0.38; 1.17]	0.8
Number of mitosis [4; 10]	[4; 10] versus 0	0.76	[0.37; 1.14]	
Number of mitosis > 10	> 10 versus 0	0.81	[0.32; 1.30]	
Ulceration status	presence versus absence	2.03	[1.20; 2.86]	< 0.001
Localisation of primary tumour	mucosal versus cutaneous or acral melanoma	1.29	[−0.06; 2.64]	0.7

### 3.6 Survival

**FIGURE 2:**
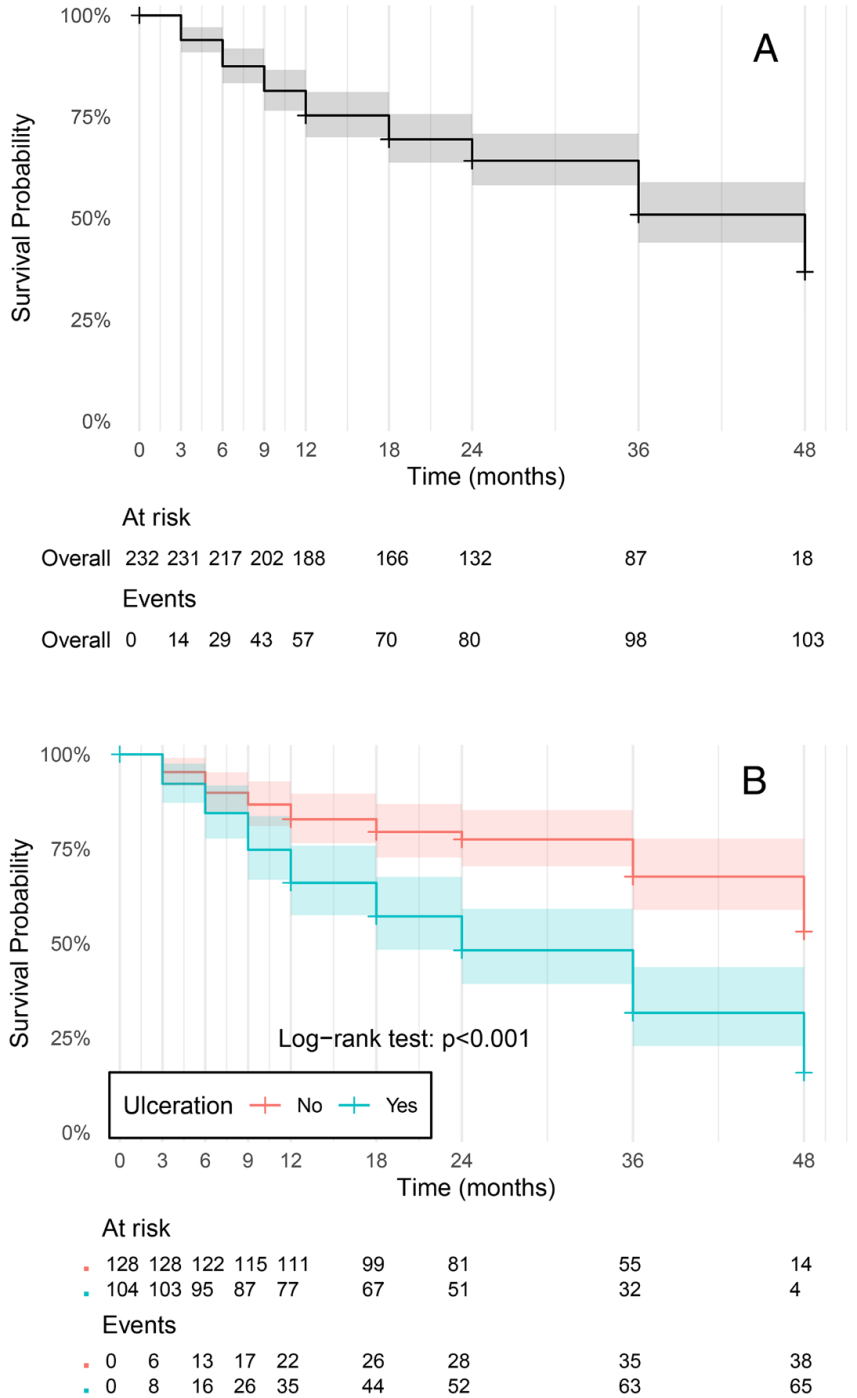
(A) Kaplan–Meier estimate of recurrence‐fee survival in study population. (B) Kaplan–Meier estimate of recurrence‐free survival according to ulceration status.

RFS at 12 months was estimated at 75% [70–81]. At 24 months, RFS was 64% [58–71] and gradually decreased to 51% at 36 months and to 37% [27–51] at 48 months of follow‐up (Figure 2A). A significant difference in RFS according to the ulceration status was observed, with a RFS of 83% [77–90] at 12 months in the patients without ulceration compared to 66% [57–76] in those with ulceration. This trend persisted at 36 months, with a RFS of 68% [59–78] in patients without ulceration compared to 32% [23–44] in those with ulceration (Figure 2B).

**FIGURE 3:**
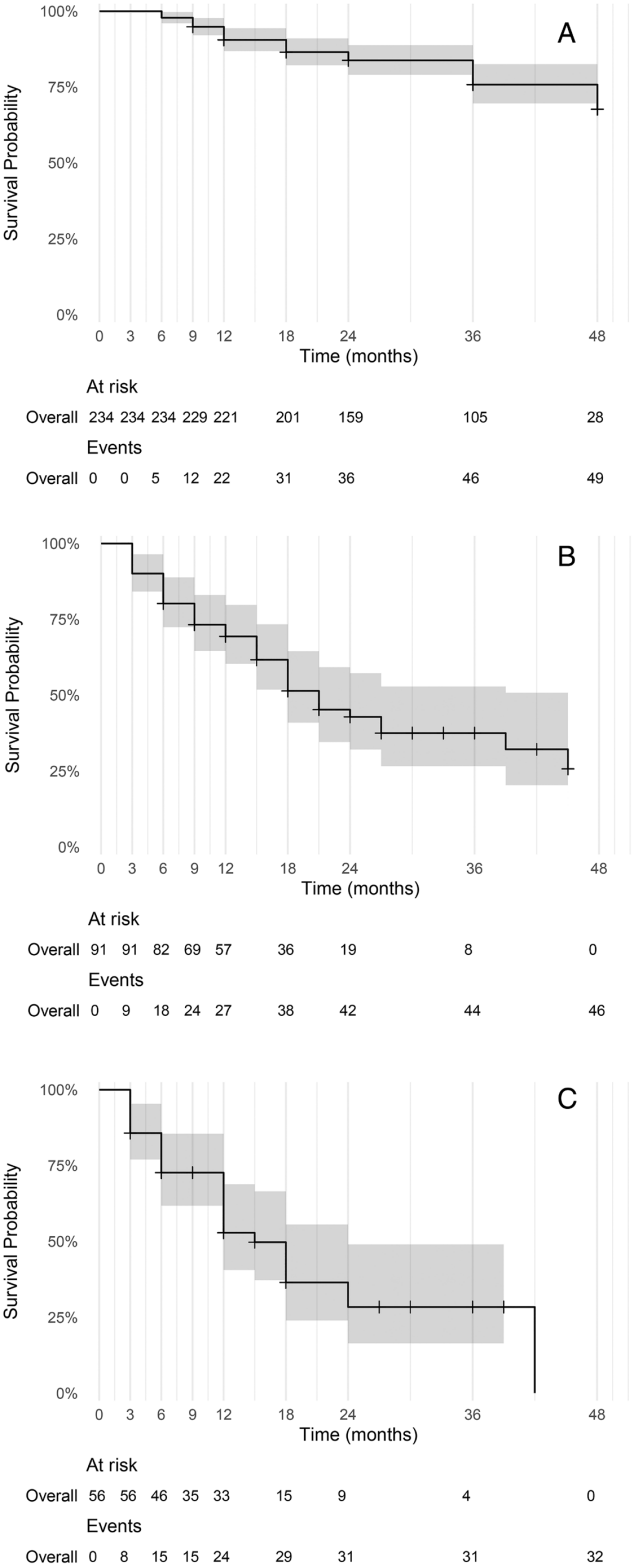
(A) Kaplan–Meier estimate of overall survival in the study population since first infusion. (B) Kaplan–Meier estimate of overall survival in the study population since first recurrence. (C) Kaplan–Meier estimate of overall survival in the study population since second recurrence.

The median OS was not reached at 48 months of follow‐up. OS was estimated at 91% [87%–94%] at 12 months, 84% [79%–89%] at 24 months, 76% [70%–83%] at 36 months and 68% [58%–79%] at 48 months (Figure 3A). The OS since first recurrence was estimated at 69% [60%–80%] at 12 months. It subsequently decreased to 43% [32%–57%] at 24 months and further declined to 37% at 36 months [27%–53%]. The median OS after the first recurrence was 21 months [18–45 months] (Figure 3B). After a second recurrence, the OS was estimated at 53% [41%–69%] at 12 months and further decreased to 28% [16%–49%] at 24 months. The median OS after the second recurrence was 15 months [12–24 months] (Figure 3C).

## 4 Discussion

The present observational study is based on real‐life data obtained from 235 patients with resected high‐risk stage III/IV melanoma treated with adjuvant anti‐PD1 immunotherapy, allowing to describe the outcomes of these patients and the clinical and laboratory features associated with recurrence in routine clinical practice.

Both the 12‐month and 24‐month RFS rates in the present cohort were in line with those previously reported in randomised trials and prospective studies [3, 4, 6, 7, 10, 11]. In addition, the RFS rates found herein confirm previous real‐world data [12, 13], highlighting that real‐life RFS is comparable to that of phase 3 trials, further demonstrating the effectiveness of adjuvant anti‐PD1 immunotherapy in resected stage III and IV melanoma. Of note, although the OS observed herein were also in line with those observed in prospective trials, these trials have not yet shown a superiority of adjuvant anti‐PD1 immunotherapy compared to placebo or anti‐CTLA‐4 immunotherapy, as ipilimumab remains, to date, the only adjuvant therapy that has shown a statistically significant increase in OS [5, 7, 14].

In terms of safety, the overall proportion of treatment‐related adverse events in the present cohort was largely below that of the two pivotal randomised trials (77.8% of treatment‐related adverse event of any grade in the EORTC 1325/KEYNOTE‐054 study and 85% in the CheckMate trial) [3, 4]. Similarly, the occurrence of vitiligo, a prognostic indicator of immunotherapy response in advanced melanoma [15, 16], was found in less than 5% of patients, a result in line with those previously reported in patients under adjuvant immunotherapy [3, 13, 17]. These discrepancies may be attributed to the underreporting of adverse events in the real‐world setting, partly due to less stringent criteria compared to interventional studies as well as a potential downplay from the medical team regarding adverse events, particularly for those of low‐grade (grade I or II) that typically have no therapeutic consequence. However, the proportion of immunotherapy discontinuation due to adverse events, a more reliable and clinically relevant indicator of tolerability, was observed in less than one‐tenth of the patients, thereby confirming the previously demonstrated tolerability of adjuvant PD‐1 immunotherapy [3, 4].

Conversely to previous reports describing a predominance of distant first recurrences [18, 19, 20], the first recurrences in the present cohort were mainly locoregional. This is likely explained by a difference in the classification of recurrences, as we chose herein to distinguish distant recurrences according to the presence or absence of cerebral involvement due to the poor prognosis associated with cerebral metastases. Herein, recurrences occurred in nearly half of the patients, mainly during treatment, a finding consistent with published observations [13, 18, 19]. This contrasts with the timing of recurrences in patients treated by adjuvant targeted therapy, which are mainly observed after the end of treatment [21].

Moreover, the analysis of second recurrence data shows nearly two‐thirds of locoregional recurrences following an initial locoregional recurrence, despite the majority of these first recurrences being managed by surgical intervention. The fact that surgery alone was not sufficient to prevent subsequent locoregional recurrences in many cases suggests that surgical treatment may not be adequate for long‐term disease control in these patients. This underscores the need to consider additional or alternative therapeutic strategies beyond surgery to improve the durability of treatment response.

Furthermore, the timing of recurrences is worth emphasising, since the majority of recurrences occurred while patients were still receiving immunotherapy, a finding consistent with the existing literature. This observation underscores the critical need for intensive monitoring during the initial months of treatment and suggests that early intervention strategies may be necessary to address potential resistance. Additionally, longer treatment‐free intervals have been shown to be generally associated with better outcomes when rechallenging with immunotherapy, further highlighting the significance of timing in the overall treatment planning [22].

Ulceration was the only characteristic found to be associated with an increased risk of recurrence and a decrease in RFS. Given the poor prognosis of patients with a first recurrence found herein, these clinical and laboratory characteristics should be taken into account when assessing the overall risk of recurrence in a patient.

This contrasts with the trend observed in the EORTC trial, in which a higher RFS was observed in ulcerated melanomas (hazard ratio 0.52) when compared to non‐ulcerated melanomas (hazard ratio 0.69). This was the case despite a comparable ulceration rate between the present cohort and the EORTC trial (40.5%). However, in this trial, only the impact of pembrolizumab against placebo was studied, which implies that there might be an increased benefit in using adjuvant immunotherapy for patients with ulcerated melanoma as these patients initially have a more unfavourable prognosis than those without ulceration. Nevertheless, the strong association between ulceration and recurrence found herein underlines that their initial unfavourable prognosis will expose them to a higher risk of later recurrence, despite having received adjuvant immunotherapy.

The hypothesis that ulceration is associated with a higher risk of recurrence is supported by the work of De Falco et al. [21], who have already demonstrated a superior RFS in patients without ulceration under adjuvant treatment (HR at 0.26). However, their study included only 30 recurrences and evaluated both adjuvant immunotherapy and targeted therapy. In another multicentre study with 1198 patients, including 1003 treated with adjuvant immunotherapy, they demonstrated that ulceration, along with other variables such as tumour stage, significantly increased the risk of recurrence (HR 1.889 [1.452; 2.458]). However, none of these variables, including ulceration, were significantly correlated with 12‐month progression‐free survival [19]. Nevertheless, since the difference in survival curves according to ulceration increased over time herein, it is possible that a 12‐month follow‐up was not enough to observe such correlations.

Age and Breslow thickness were the only two factors found to be significantly associated with recurrence rate in the univariate analysis but they did not remain statistically significant in the multivariate analysis [10]. Although other known prognostic factors for melanoma, such as mitotic rate and mutational status, were included in the present analysis, they did not emerge as significant contributors in the multivariate analysis. The lack of statistical significance for these variables may be due to the limited sample size or the inherent variability of real‐world data. Additionally, the absence of a detailed analysis of certain factors, such as immunohistochemical characteristics, underscores the need for future studies with larger sample sizes and more targeted analyses to better understand the mechanisms underlying recurrences.

The present study has limitations, mainly related to its retrospective design and the relatively limited number of patients included. Further prospective studies, with larger sample sizes, including notably a control group comprising patients under observation only after surgery would help reinforce the conclusion regarding the role of ulceration as a predictive factor of recurrence. Although such prospective studies are needed to validate the present findings, it is important to note that the survival data obtained after a long follow‐up were similar to those of pivotal clinical trials and other real‐life studies, reinforcing the effectiveness of adjuvant anti‐PD1 therapy in real life.

## 5 Conclusion

The present study provides a comprehensive view of the outcomes and factors associated with recurrence in patients with resected high‐risk stage III/IV melanoma. Ulceration emerged as the only significant predictive factor for recurrence and was associated with a decrease in recurrence‐free survival at 24 months. The clinical implications of these findings could ultimately guide therapeutic decisions and enhance follow‐up strategies.

## Author Contributions

**Liza Benzoni:** conceptualization (equal), data curation (equal), investigation (lead), methodology (equal), writing – original draft (lead). **Anaïs Eberhardt:** data curation (equal), project administration (supporting), writing – review and editing (equal). **Sarah Milley:** data curation (equal), writing – review and editing (equal). **Safa Idoudi:** conceptualization (equal), writing – review and editing (equal). **Camille Trefcon:** data curation (equal), writing – review and editing (equal). **Nicolas Romain‐Scelle:** formal analysis (lead), software (lead), writing – review and editing (equal). **Luc Thomas:** writing – review and editing (equal). **Stéphane Dalle:** conceptualization (lead), project administration (lead), supervision (lead), writing – review and editing (lead).

## Ethics Statement

The study was approved by the Scientific and Ethical Committee of the *Hospices Civils de Lyon* (CSE‐HCL IRB 00013204) on the 9th of September 2022 (opinion n°22_616). A waiver/exempt was granted by the IRB/Ethics Committee for written consent by human participants. The use of personal data followed the French Reference Methodology MR‐004 of the national data protection agency (*Commission Nationale de l'Informatique et des Libertés*, CNIL).

## Conflicts of Interest

Stéphane Dalle received institutional research grant from MSD, BMS, Pierre‐Fabre. Stéphane Dalle: spouse Sanofi employee.

## Data Availability

The data underlying this article are available in the article and in its online Supporting Information.
